# Combined Associations of Liver Enzymes and Obesity With Diabetes Mellitus Prevalence: The Tohoku Medical Megabank Community-based Cohort Study

**DOI:** 10.2188/jea.JE20200384

**Published:** 2022-05-05

**Authors:** Fumi Itabashi, Takumi Hirata, Mana Kogure, Akira Narita, Naho Tsuchiya, Tomohiro Nakamura, Naoki Nakaya, Ryohei Sasaki, Nobuyuki Takanashi, Kiyomi Sakata, Kozo Tanno, Junichi Sugawara, Shinichi Kuriyama, Ichiro Tsuji, Shigeo Kure, Atsushi Hozawa

**Affiliations:** 1 Tohoku Medical Megabank Organization, Tohoku University, Sendai, Japan; 2 Hokkaido University Faculty of Medicine, Sapporo, Japan; 3 Saitama Prefectural University, Saitama, Japan; 4 Department of Human Sciences, Center for Liberal Arts and Sciences, Iwate Medical University, Iwate, Japan; 5 Iwate Tohoku Medical Megabank Organization, Disaster Reconstruction Center, Iwate Medical University, Iwate, Japan

**Keywords:** diabetes mellitus, alanine aminotransferase, gamma-glutamyl transferase, obesity, non-obesity

## Abstract

**Background:**

Alanine aminotransferase (ALT) and gamma-glutamyl transferase (GGT) are enzymes associated with diabetes mellitus (DM) prevalence. However, limited information is available regarding the association of liver enzymes and DM consistently present in obese and non-obese individuals. We examined whether the combination of ALT and GGT enzymes is associated with the prevalence of DM, regardless of obesity, in a general Japanese population.

**Methods:**

We conducted a cross-sectional study of 62,786 participants aged ≥20 years who lived in Miyagi and Iwate, Japan. We divided all the participants into eight groups according to the ALT level (low: <30 IU/L and high: ≥30 IU/L), GGT level (low: <50 IU/L and high: ≥50 IU/L), and the presence of obesity. We calculated odds ratios (ORs) and 95% confidence intervals (CIs) using multivariable logistic regression analysis, adjusting for potential confounders, to determine associations of the combination of ALT and GGT levels and obesity with DM prevalence.

**Results:**

Overall, 6,008 participants (9.6%) had DM. Compared to non-obese individuals with low ALT and GGT levels, the participants with high ALT and GGT levels had high ORs for DM in both obese (OR 4.06; 95% CI, 3.61–4.56) and non-obese groups (OR 2.19; 95% CI, 1.89–2.52). The obese group had high ORs for DM, even at low ALT and GGT levels.

**Conclusion:**

High ALT and GGT levels are associated with DM prevalence in obese and non-obese participants. This finding suggests that correcting ALT and GGT levels and controlling obesity are important for the prevention of DM.

## INTRODUCTION

Obesity is a well-known risk factor for the development of diabetes mellitus (DM).^1^^–^^3^ Accordingly, screening for obesity is an appropriate approach to distinguish between the participants with high and low risk. However, previous epidemiological studies in the Asian and Japanese populations have reported that the prevalence of DM is increasing even in non-obese individuals.^4^^–^^6^ Therefore, screening for obesity alone may overlook people at high risk of DM. Thus, a method for screening high risk individuals with DM other than screening for obesity is expected to be established.

Previous epidemiological studies in the Japanese population have reported that fatty liver is associated with the development of DM, regardless of obesity.^7^^,^^8^ Therefore, we considered it appropriate to first screen non-obese individuals with fatty liver to detect the risk of DM. However, fatty liver is commonly diagnosed by ultrasonography and liver biopsy. These diagnostic evaluations are expensive, time-consuming, and have a higher risk of complications. Therefore, they are not suitable for health screenings.

Alanine aminotransferase (ALT) and gamma-glutamyl transferase (GGT) enzymes are surrogate markers of fatty liver diseases.^9^^,^^10^ ALT and GGT tests are blood tests used as liver function indicators during health examinations, and are relatively inexpensive. Previous studies have reported that ALT and GGT levels are associated with the development of DM.^11^^–^^19^ However, few reports have evaluated the association of ALT and GGT levels with DM in non-obese individuals.

Therefore, we aimed to evaluate whether the combination of ALT and GGT levels is associated with the prevalence of DM among obese and non-obese individuals.

## METHODS

### Study participants

This cross-sectional study was conducted to evaluate the relationship between the combination of ALT and GGT levels and the prevalence of DM in obese and non-obese individuals. Participants who fulfilled the following criteria were included in this study: (1) those who participated in the baseline survey from the Tohoku Medical Megabank Community-Based Project Cohort Study (TMM CommCohort Study),^20^ (2) those who were aged ≥20 years and were living in Miyagi Prefecture and Iwate Prefecture between May 2013 and March 2016; and (3) those who joined the TMM CommCohort Study during the municipal health checkup. The TMM CommCohort Study is a population-based prospective cohort study that has been ongoing since 2013. Written informed consent was obtained from each participant. This study was approved by the Institutional Review Board of the Tohoku Medical Megabank Organization (approval number: 2019-4-065).

In total, 66,283 participants were initially included in the baseline survey of the municipal health checkup. However, we excluded 3,497 participants for the following reasons: (1) lack of self-reported questionnaire (*n* = 3,259), (2) incomplete self-reported questionnaire (*n* = 97), (3) missing data on body weight, height, ALT and GGT levels, hemoglobin A1c (HbA1c) level, blood glucose level, systolic blood pressure (SBP), and diastolic blood pressure (DBP) (*n* = 141). Finally, we analyzed the data for 62,786 participants.

### Measurements

We analyzed the participants who completed a self-reported questionnaire to assess demographic characteristics, body weight, height, smoking status, alcohol drinking status, self-reported family history of DM, information on treatment for DM, information on treatment for hypertension, self-reported history of hepatitis B and hepatitis C, and self-reported leisure-time physical activity. The body mass index (BMI) was calculated as the weight (kg) divided by the square of the height (m). Obesity was defined as BMI ≥25 kg/m^2^ based on the Western Pacific Region of World Health Organization criteria for Japanese individuals.^21^ The participants were divided into two groups based on BMI ≥25 kg/m^2^: obese and non-obese. If the participants reported a family history of type 2 DM (ie, father, mother, brother, or sister with DM), we defined it as a family history of DM.

Smoking status (number of cigarettes per day) was determined using a self-administered questionnaire. First, we classified the participants into three categories: never smokers, ex-smokers, and current smokers. Participants who reported not smoking more than 100 cigarettes in their lifetime were classified as never smokers. Participants who reported having smoked 100 or more cigarettes over their lifetime and who were currently smoking were classified as current smokers. Current smokers were further classified into the following three categories: ≤10 per/day, >10 per and ≤20 per/day, >20 per/day. The participants who reported having smoked more than 100 cigarettes during their lifetime and who were currently not smoking were classified as ex-smokers. Thus, we classified smoking status as follows: never smoker, ex-smoker, current ≤10 per/day, >10 per and ≤20 per/day, and >20 per/day. Missing smoking status data were categorized as a missing group.

Alcohol drinking status (frequency and amount per day) was determined using a self-administered questionnaire, and the participants were divided into four categories: current drinkers, ex-drinkers, never drinkers, and cannot drink constitutionally. The type of alcohol was classified into the following six categories: sake, distilled spirits, shochu-based beverages, beer, whiskey, and wine. The frequency of alcohol intake was classified into the following six categories: almost never, 1–3 days/month, 1–2 days/week, 3–4 days/week, 5–6 days/week, and daily. The participants answered how much of each type of alcohol they drank. Each type of alcohol intake was multiplied by the frequency and amount and converted to the amount of ethanol. The amount of alcohol consumption was classified into the following four categories: <23 g/day, ≥23 g and <46 g/day, ≥46 g and <69 g/day, ≥69 g/day. Thus, we classified alcohol drinking status as follows: never drinker, ex-drinker, current <23 g/day, ≥23 g and <46 g/day, ≥46 g and <69 g/day, and ≥69 g/day. Missing alcohol drinking status data were categorized as a missing group. The participants were asked the average frequency (times/week) and duration (min/time) of normal walking, brisk walking, moderate-intensity exercise, hard-intensity exercise. Metabolic equivalents (METs) assigned to each physical activity were used to quantify the amount of leisure-time physical activity.^22^ We used the quartile of physical activities in our model; missing data were categorized as a missing group.

Blood samples were collected at the venues of the municipal health checkup. Although the participants were instructed to participate in the fasting condition, some participants did not fulfill this criterion. Plasma glucose concentrations and HbA1c levels were analyzed using an enzymatic method. The presence of DM was defined as plasma glucose ≥200 mg/dL and/or HbA1c ≥6.5% and/or receiving treatment for DM.^23^ ALT and GGT levels were measured using an enzymatic method. Liver dysfunction was defined as ALT >30 IU/L and GGT >50 IU/L based on health examinations in Japan. Triglycerides (TG) level was measured using an enzymatic method. We defined hypertriglyceridemia as TG level ≥150 mg/dL. Low-density lipoprotein (LDL) and high-density lipoprotein (HDL) levels were measured using a direct method. We defined high LDL as LDL ≥120 mg/dL. Missing LDL data were categorized as a missing group. We defined low HDL as HDL <40 mg/dL. SBP and DBP were measured with an automatic sphygmomanometer. The presence of hypertension was defined as SBP ≥140 mm Hg and/or DBP ≥90 mm Hg and/or receiving treatment for hypertension.^24^ The area was divided into Miyagi Prefecture and Iwate Prefecture.

### Statistical analysis

First, the participants were categorized into four groups according to the ALT level (low: <30 IU/L and high: ≥30 IU/L) and GGT level (low: <50 IU/L and high: ≥50 IU/L) as follows: (1) low ALT and GGT levels; (2) low ALT and high GGT levels; (3) high ALT and low GGT levels; and (4) high ALT and GGT levels. Next, the participants were categorized into eight groups according to the ALT level, GGT level, and presence of obesity (non-obesity: <25 kg/m^2^ and obesity: ≥25 kg/m^2^) as follows: (1) non-obese, low ALT and GGT levels; (2) non-obese, low ALT and high GGT levels; (3) non-obese, high ALT and low GGT levels; (4) non-obese, high ALT and GGT levels; (5) obese, low ALT and GGT levels; (6) obese, low ALT and high GGT levels; (7) obese, high ALT and low GGT levels; and (8) obese, high ALT and GGT levels.

We used analysis of variance or the Kruskal-Wallis test for continuous variables and the chi-square test for categorical variables to compare the characteristics of the combination of ALT and GGT levels in the four groups. A similar analysis was performed in eight groups considering the presence or absence of obesity. The data are presented as means (standard deviations) or medians (interquartile ranges) for continuous variables, and as numbers (percentages) for categorical variables.

Multivariable logistic regression models were used to obtain odds ratios (ORs) and 95% confidence intervals (CIs) to assess the combined associations of the ALT and GGT levels with the prevalence of DM. The models were adjusted for age, sex, BMI, smoking status, alcohol drinking status, family history of diabetes, hypertriglyceridemia, high LDL, low HDL, hypertension, physical activity, and area. Eight groups of models were adjusted for age, sex, smoking status, alcohol drinking status, family history of diabetes, hypertriglyceridemia, high LDL, low HDL, hypertension, physical activity, and area, excluding BMI. We further performed a stratified analysis. We stratified our participants using the following variables: sex (male and female), alcohol drinking status (never drinker, ex-drinker and current drinker), and residential areas (Miyagi Prefecture and Iwate Prefecture). We also performed an analysis excluding participants with a history of hepatitis C or hepatitis B.

Two-tailed *P* values <0.05 were considered statistically significant. All analyses were performed using the Statistical Analysis System software, version 9.4 for Windows (SAS Inc., Cary, NC, USA).

## RESULTS

The baseline characteristics of all participants according to the combination of ALT and GGT levels are shown in Table 1. We analyzed data from 62,786 participants (23,564 male and 39,222 female). The results showed that 6,008 individuals (3,398 male and 2,610 female, 9.6%) had DM. The prevalence of DM was highest in the group with high ALT and GGT levels, compared to the group with both low ALT and GGT levels. Obesity and BMI were higher among the high ALT level groups. The proportions of current smokers and current drinkers were higher among the high GGT level groups.

**Table 1.** The baseline characteristics of all participants according to the combination of ALT and GGT levels

	All		Low ALT Low GGT		Low ALT High GGT		High ALT Low GGT		High ALT High GGT		*P* value^c^
Number of participants	62,786		48,450		5,080		4,950		4,306		
Residential areas, *n* (%)											
Miyagi	37,064	(59.0)	28,988	(59.8)	2,830	(55.7)	2,868	(57.9)	2,378	(55.2)	<0.001
Iwate	25,722	(41.0)	19,462	(40.2)	2,250	(44.3)	2,082	(42.1)	1,928	(44.8)	
Male sex, *n* (%)	23,564	(37.5)	14,430	(29.8)	3,514	(69.2)	2,497	(50.4)	3,123	(72.5)	<0.001
Age, years	60.8	(11.0)	60.7	(11.3)	62.8	(8.8)	61.1	(10.7)	59.3	(10.7)	<0.001
BMI, kg/m^2^	23.5	(3.5)	23.0	(3.3)	24.0	(3.2)	25.7	(3.9)	25.8	(3.9)	<0.001
Number of obesity (BMI ≥25 kg/m^2^), *n* (%)	18,606	(29.6)	11,705	(24.2)	1,768	(34.8)	2,758	(55.7)	2,375	(55.2)	<0.001
ALT, IU/L	18	(14–25)	17	(13–21)	22	(18–26)	37	(33–46)	44	(36–59)	<0.001
GGT, IU/L	22	(16–36)	19	(15–26)	71	(58–96)	31	(23–39)	87	(65–138)	<0.001
Diabetes,^a^ *n* (%)	6,008	(9.6)	3,674	(7.6)	615	(12.1)	865	(17.5)	854	(19.8)	<0.001
HbA1c, %	5.7	(0.6)	5.6	(0.5)	5.7	(0.7)	5.9	(0.7)	5.9	(0.9)	<0.001
Non-fasting plasma glucose, mg/dL	93	(85–105)	92	(84–103)	97	(89–111)	97	(88–110)	100	(90–116)	<0.001
Medication for diabetes, *n* (%)	4,074	(6.5)	2,690	(5.6)	384	(7.6)	553	(11.2)	447	(10.4)	<0.001
Family history of diabetes, *n* (%)	5,997	(9.6)	4,621	(9.5)	407	(8.0)	525	(10.6)	444	(10.3)	<0.001
Hypertension,^b^ *n* (%)	25,229	(40.2)	17,663	(36.5)	2,757	(54.3)	2,447	(49.4)	2,362	(54.9)	<0.001
SBP, mm Hg	126.2	(17.3)	125.0	(17.2)	130.8	(17.3)	129.0	(16.5)	131.4	(16.3)	<0.001
DBP, mm Hg	75.5	(10.4)	74.5	(10.3)	78.8	(10.5)	77.5	(10.0)	80.1	(10.1)	<0.001
Medication for hypertension, *n* (%)	16,973	(27.0)	11,812	(24.4)	1,864	(36.7)	1,727	(34.9)	1,570	(36.5)	<0.001
TG, mg/dL	101	(72–147)	94	(68–134)	125	(87–185)	125	(87–179)	152	(105–226)	<0.001
Hypertriglyceridemia (TG ≥150 mg/dL), *n* (%)	15,162	(24.2)	9,266	(19.1)	1,901	(37.4)	1,787	(36.1)	2,208	(51.3)	<0.001
HDL cholesterol, mg/dL	63.1	(16.3)	64.2	(16.0)	63.2	(16.6)	56.0	(14.9)	57.9	(16.6)	<0.001
Low HDL (HDL <40 mg/dL), *n* (%)	2,968	(4.7)	1,826	(3.8)	220	(4.3)	503	(10.2)	419	(9.7)	<0.001
LDL cholesterol (*N* = 58,063), mg/dL	121.6	(30.6)	121.9	(29.8)	117.2	(32.5)	123.2	(31.4)	121.9	(35.4)	<0.001
High LDL (LDL ≥120 mg/dL), *n* (%)	29,144	(50.2)	22,429	(50.6)	2,175	(44.5)	2,439	(52.2)	2,101	(50.7)	<0.001
Smoking status (*N* = 61,636), *n* (%)											
Never smoker	38,446	(62.4)	32,176	(67.8)	1,854	(36.9)	2,867	(58.9)	1,549	(36.3)	<0.001
Ex-smoker	14,465	(23.5)	9,693	(20.4)	1,855	(36.9)	1,343	(27.6)	1,574	(36.9)	
Current smoker (≤10 number cigarettes per day)	2,786	(4.5)	2,039	(4.3)	289	(5.8)	190	(3.9)	268	(6.3)	
Current smoker (>10 and ≤20 number cigarettes per day)	4,879	(7.9)	3,005	(6.3)	808	(16.1)	391	(8.0)	675	(15.8)	
Current smoker (>20 number cigarettes per day)	1,060	(1.7)	566	(1.2)	220	(4.4)	76	(1.6)	198	(4.6)	
Drinking status (*N* = 62,253), *n* (%)											
Never drinker	29,838	(47.9)	25,162	(52.4)	1,026	(20.3)	2,586	(52.6)	1,064	(24.8)	<0.001
Ex-drinker	1,799	(2.9)	1,360	(2.8)	113	(2.2)	205	(4.2)	121	(2.8)	
Current drinker (<23 g/day)	18,479	(29.7)	14,807	(30.9)	1,127	(22.3)	1,451	(29.5)	1,094	(25.5)	
Current drinker (≥23 g and <46 g/day)	6,372	(10.2)	4,017	(8.4)	1,150	(22.8)	406	(8.3)	799	(18.7)	
Current drinker (≥46 g and <69 g/day)	3,075	(4.9)	1,567	(3.3)	787	(15.6)	174	(3.5)	547	(12.8)	
Current drinker (≥69 g/day)	2,690	(4.3)	1,082	(2.3)	852	(16.9)	98	(2.0)	658	(15.4)	
Hepatitis B, *n* (%)	941	(1.5)	699	(1.4)	79	(1.6)	88	(1.8)	75	(1.7)	0.140
Hepatitis C, *n* (%)	485	(0.8)	330	(0.7)	34	(0.7)	75	(1.5)	46	(1.1)	<0.001
Leisure-time physical activity (*N* = 62,275)											
METs	63.0	(6.8–198.0)	66.3	(8.4–210.0)	57.9	(2.8–183.9)	57.9	(0–183.9)	39.0	(0–144.0)	<0.001

ALT, alanine aminotransferase; BMI, body mass index; DBP, diastolic blood pressure; GGT, gamma-glutamyl transferase; HbA1c, hemoglobin A1c; HDL, high-density lipoprotein; LDL, low-density lipoprotein; METs, Metabolic equivalents; SBP, systolic blood pressure; TG, triglycerides.

The values in the table are means (standard deviations) or medians (interquartile ranges) for continuous variables and numbers (percentage) for categorical variables.

^a^Diabetes was defined as plasma glucose ≥200 mg/dL and/or HbA1c ≥6.5% and/or receiving treatment for diabetes.

^b^Hypertension was defined as SBP ≥140 mm Hg and/or DBP ≥90 mm Hg and/or receiving treatment for hypertension.

^c^Analysis of variance or the Kruskal-Wallis test for continuous variables and the chi-square test for categorical variables.

The baseline characteristics of obese and non-obese participants according to the combination of ALT and GGT levels are shown in Table 2. The prevalence of DM was highest in the obese group of participants with high levels of ALT and GGT than in the non-obese group with both low ALT and GGT levels. In non-obese individuals, groups with high ALT and GGT levels had a high prevalence of DM. Moreover, the obese group comprised a higher proportion of individuals with DM, even in the subgroups of participants with both low ALT and GGT levels, than the non-obese group of participants with both low ALT and GGT levels. The proportions of current smokers and current drinkers were higher among the high GGT level groups, regardless of obesity.

**Table 2.** The baseline characteristics of obese and non-obese participants according to the combination of ALT and GGT levels

	All		Non-obesity (BMI <25 kg/m^2^)								Obesity (BMI ≥25 kg/m^2^)								*P* value^c^
	
			Low ALT Low GGT		Low ALT High GGT		High ALT Low GGT		High ALT High GGT		Low ALT Low GGT		Low ALT High GGT		High ALT Low GGT		High ALT High GGT		
Number of participants	62,786		36,745		3,312		2,192		1,931		11,705		1,768		2,758		2,375		
Residential areas, *n* (%)																			
Miyagi	37,064	(59.0)	22,266	(60.6)	1,879	(56.7)	1,293	(59.0)	1,061	(55.0)	6,722	(57.4)	951	(53.8)	1,575	(57.1)	1,317	(55.5)	<0.001
Iwate	25,722	(41.0)	14,479	(39.4)	1,433	(43.3)	899	(41.0)	870	(45.1)	4,983	(42.6)	817	(46.2)	1,183	(42.9)	1,058	(44.6)	
Male sex, *n* (%)	23,564	(37.5)	10,467	(28.5)	2,256	(68.1)	1,121	(51.1)	1,395	(72.2)	3,963	(33.9)	1,258	(71.2)	1,376	(49.9)	1,728	(72.8)	<0.001
Age, years	60.8	(11.0)	60.2	(11.6)	62.6	(9.0)	61.6	(10.4)	60.2	(10.0)	62.4	(10.1)	63.2	(8.6)	60.7	(10.9)	58.6	(11.2)	<0.001
BMI, kg/m^2^	23.5	(3.5)	21.6	(2.1)	22.2	(2.0)	22.4	(2.0)	22.6	(1.8)	27.5	(2.4)	27.4	(2.2)	28.3	(2.9)	28.4	(3.1)	<0.001
ALT, IU/L	18	(14–25)	16	(13–20)	22	(18–26)	36	(33–43)	41	(34–52)	19	(15–23)	23	(19–27)	38	(34–48)	46	(37–65)	<0.001
GGT, IU/L	22	(16–36)	18	(14–25)	71	(59–96)	29	(22–37)	97	(68–166)	22	(17–30)	70	(58–96)	32	(25–40)	82	(64–122)	<0.001
Diabetes,^a^ *n* (%)	6,008	(9.6)	2,145	(5.8)	320	(9.7)	296	(13.5)	289	(15.0)	1,529	(13.1)	295	(16.7)	569	(20.6)	565	(23.8)	<0.001
HbA1c, %	5.7	(0.6)	5.6	(0.5)	5.6	(0.7)	5.7	(0.7)	5.7	(0.8)	5.8	(0.6)	5.8	(0.7)	6.0	(0.8)	6.0	(0.9)	<0.001
Non-fasting plasma glucose, mg/dL	93	(85–105)	91	(84–101)	96	(87–109)	94	(86–106)	97	(88–112)	95	(87–108)	100	(90–116)	98	(89–113)	101	(91–119)	<0.001
Medication for diabetes, *n* (%)	4,074	(6.5)	1,579	(4.3)	194	(5.9)	202	(9.2)	164	(8.5)	1,111	(9.5)	190	(10.8)	351	(12.7)	283	(11.9)	<0.001
Family history of diabetes, *n* (%)	5,997	(9.6)	3,516	(9.6)	252	(7.6)	224	(10.2)	178	(9.2)	1,105	(9.4)	155	(8.8)	301	(10.9)	266	(11.2)	<0.001
Hypertension,^b^ *n* (%)	25,229	(40.2)	11,362	(30.9)	1,593	(48.1)	840	(38.3)	954	(49.4)	6,301	(53.8)	1,164	(65.8)	1,607	(58.3)	1,408	(59.3)	<0.001
SBP, mm Hg	126.2	(17.3)	123.3	(17.1)	129.5	(17.5)	125.5	(16.4)	130.0	(16.7)	130.3	(16.5)	133.1	(16.8)	131.9	(16.0)	132.5	(16.0)	<0.001
DBP, mm Hg	75.5	(10.4)	73.5	(10.2)	78.1	(10.5)	75.2	(9.9)	79.0	(10.1)	77.5	(9.8)	80.1	(10.3)	79.4	(9.7)	81.0	(9.9)	<0.001
Medication for hypertension, *n* (%)	16,973	(27.0)	7,235	(19.7)	1,010	(30.5)	565	(25.8)	591	(30.6)	4,577	(39.1)	854	(48.3)	1,162	(42.1)	979	(41.2)	<0.001
TG, mg/dL	101	(72–147)	89	(65–125)	117	(82–174)	109	(76–156)	139	(93–212)	115	(84–161)	141	(101–201)	138	(100–194)	162	(116–236)	<0.001
Hypertriglyceridemia (TG ≥150 mg/dL), *n* (%)	15,162	(24.2)	5,798	(15.8)	1,099	(33.2)	597	(27.2)	876	(45.4)	3,468	(29.6)	802	(45.4)	1,190	(43.2)	1,332	(56.1)	<0.001
HDL cholesterol, mg/dL	63.1	(16.3)	66.3	(16.2)	65.7	(17.2)	60.3	(16.4)	63.0	(17.9)	57.8	(13.9)	58.5	(14.3)	52.7	(12.7)	53.8	(14.2)	<0.001
Low HDL (HDL <40 mg/dL), *n* (%)	2,968	(4.7)	1,021	(2.8)	98	(3.0)	149	(6.8)	116	(6.0)	805	(6.9)	122	(6.9)	354	(12.8)	303	(12.8)	<0.001
LDL cholesterol (*N* = 58,063), mg/dL	121.6	(30.6)	121.3	(29.8)	115.6	(32.9)	121.0	(31.5)	115.0	(35.7)	123.8	(29.8)	120.1	(31.4)	125.0	(31.2)	127.4	(34.2)	<0.001
High LDL (LDL ≥120 mg/dL), *n* (%)	29,144	(50.2)	16,656	(49.8)	1,353	(42.6)	1,045	(50.5)	813	(43.9)	5,773	(53.0)	822	(48.0)	1,394	(53.6)	1,288	(56.2)	<0.001
Smoking status (*N* = 61,636), *n* (%)																			
Never smoker	38,446	(62.4)	24,611	(68.3)	1,160	(35.4)	1,243	(57.6)	640	(33.4)	7,565	(66.3)	694	(39.8)	1,624	(59.9)	909	(38.7)	<0.001
Ex-smoker	14,465	(23.5)	7,028	(19.5)	1,149	(35.0)	593	(27.5)	688	(35.9)	2,665	(23.3)	706	(40.5)	750	(27.7)	886	(37.7)	
Current smoker (≤10 number cigarettes per day)	2,786	(4.5)	1,667	(4.6)	217	(6.6)	101	(4.7)	133	(7.0)	372	(3.3)	72	(4.1)	89	(3.3)	135	(5.8)	
Current smoker (>10 and ≤20 number cigarettes per day)	4,879	(7.9)	2,353	(6.5)	597	(18.2)	184	(8.5)	354	(18.5)	652	(5.7)	211	(12.1)	207	(7.6)	321	(13.7)	
Current smoker (>20 number cigarettes per day)	1,060	(1.7)	403	(1.1)	158	(4.8)	36	(1.7)	100	(5.2)	163	(1.4)	62	(3.6)	40	(1.5)	98	(4.2)	
Drinking status (*N* = 62,253), *n* (%)																			
Never drinker	29,838	(47.9)	18,886	(51.9)	645	(19.6)	1,069	(49.0)	396	(20.6)	6,276	(54.2)	381	(21.7)	1,517	(55.4)	668	(28.3)	<0.001
Ex-drinker	1,799	(2.9)	991	(2.7)	76	(2.3)	88	(4.0)	52	(2.7)	369	(3.2)	37	(2.1)	117	(4.3)	69	(2.9)	
Current drinker (<23 g/day)	18,479	(29.7)	11,472	(31.5)	734	(22.2)	685	(31.4)	441	(22.9)	3,335	(28.8)	393	(22.4)	766	(28.0)	653	(27.7)	
Current drinker (≥23 g and <46 g/day)	6,372	(10.2)	3,066	(8.4)	759	(23.0)	198	(9.1)	401	(20.8)	951	(8.2)	391	(22.3)	208	(7.6)	398	(16.9)	
Current drinker (≥46 g and <69 g/day)	3,075	(4.9)	1,200	(3.3)	523	(15.9)	86	(3.9)	297	(15.4)	367	(3.2)	264	(15.0)	88	(3.2)	250	(10.6)	
Current drinker (≥69 g/day)	2,690	(4.3)	810	(2.2)	563	(17.1)	55	(2.5)	337	(17.5)	272	(2.4)	289	(16.5)	43	(1.6)	321	(13.6)	
Hepatitis B, *n* (%)	941	(1.5)	498	(1.4)	50	(1.5)	46	(2.1)	40	(2.1)	201	(1.7)	29	(1.6)	42	(1.5)	35	(1.5)	0.009
Hepatitis C, *n* (%)	485	(0.8)	251	(0.7)	21	(0.6)	51	(2.3)	21	(1.1)	79	(0.7)	13	(0.7)	24	(0.9)	25	(1.1)	<0.001
Leisure-time physical activity (*N* = 62,275)																			
METs	63.0	(6.8–198.0)	67.5	(8.4–216.0)	60.0	(2.8–193.5)	63.0	(2.8–208.3)	45.0	(0–166.5)	63.0	(3.0–193.5)	57.9	(2.8–180.0)	50.7	(0–173.8)	30.0	(0–135.0)	<0.001

ALT, alanine aminotransferase; BMI, body mass index; DBP, diastolic blood pressure; GGT, gamma-glutamyl transferase; HbA1c, hemoglobin A1c; HDL, high-density lipoprotein; LDL, low-density lipoprotein; METs, Metabolic equivalents; SBP, systolic blood pressure; TG, triglycerides.

The values in the table are means (standard deviations) or medians (interquartile ranges) for continuous variables and numbers (percentage) for categorical variables.

^a^Diabetes was defined as plasma glucose ≥200 mg/dL and/or HbA1c ≥6.5% and/or receiving treatment for diabetes.

^b^Hypertension was defined as SBP ≥140 mm Hg and/or DBP ≥90 mm Hg and/or receiving treatment for hypertension.

^c^Analysis of variance or the Kruskal-Wallis test for continuous variables and the chi-square test for categorical variables.

**Figure 1.**
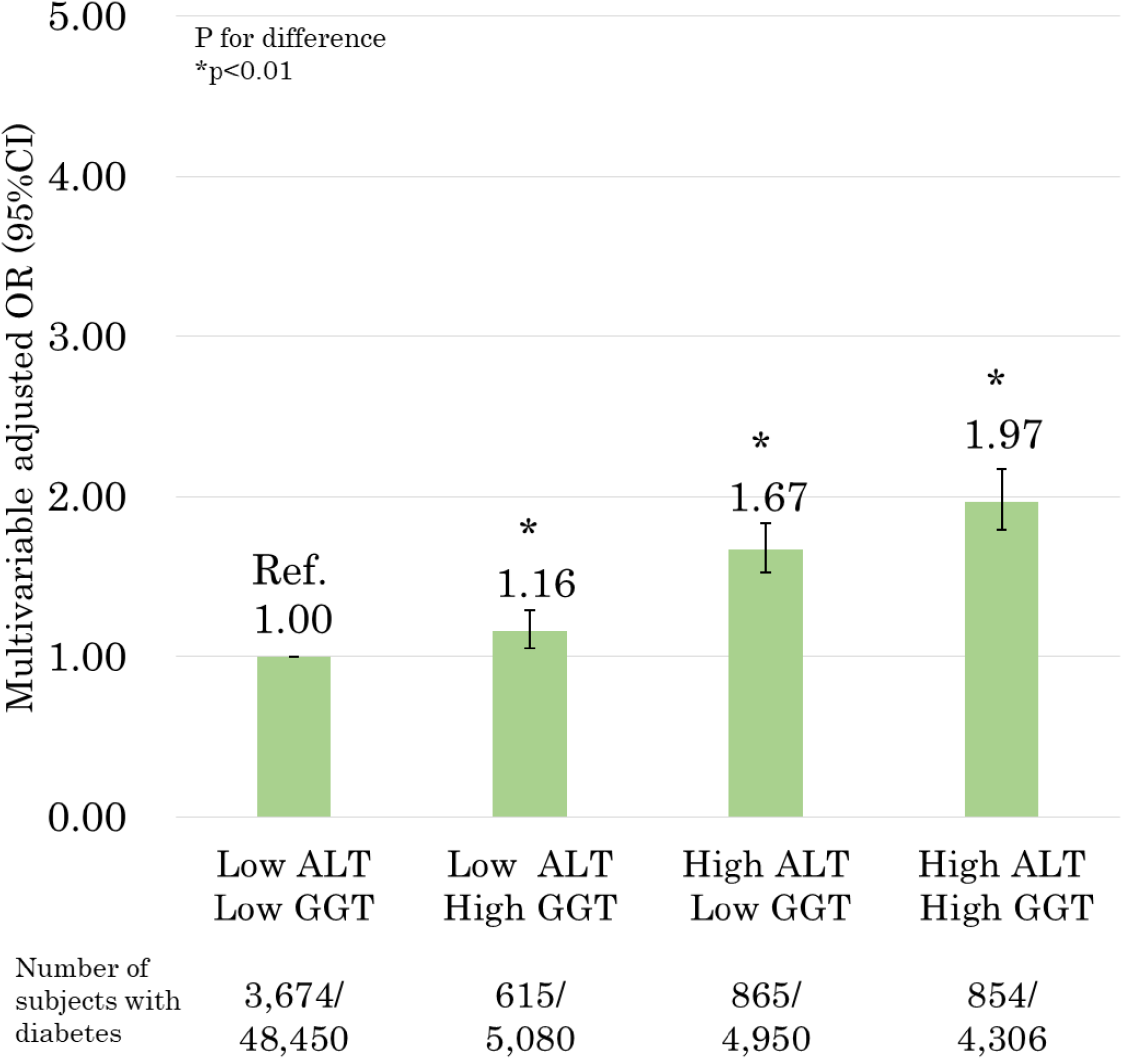
Relationship between the combination of alanine aminotransferase (ALT) and gamma-glutamyl transferase (GGT) levels with the prevalence of diabetes mellitus (DM) (adjusted for age, sex, body mass index [BMI], smoking status, alcohol drinking status, family history of diabetes, hypertriglyceridemia, high low-density lipoprotein [LDL], low high-density lipoprotein [HDL], hypertension, physical activity, and area). *P* values for difference were derived from multiple logistic regression analysis. Bars represent 95% confidence intervals.

The results of the relationship between the combination of ALT and GGT levels and the prevalence of DM are presented in Figure 1. In multivariable analysis, the group with both high ALT and GGT levels had the highest OR for the prevalence of DM, compared to the group with both low ALT and GGT levels (OR 1.97; 95% CI, 1.79–2.17). The groups with high ALT or GGT levels had significantly higher OR for prevalence of DM [low ALT and high GGT levels (OR 1.16; 95% CI, 1.05–1.29) and high ALT and low GGT levels (OR 1.67; 95% CI, 1.53–1.83)].

**Figure 2.**
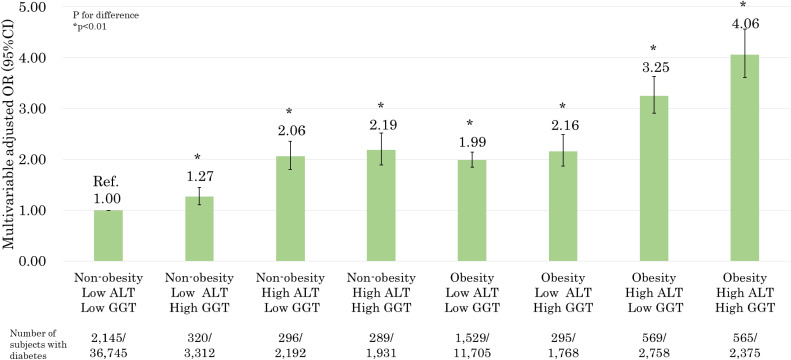
Relationship between the combination of alanine aminotransferase (ALT) and gamma-glutamyl transferase (GGT) levels with the prevalence of diabetes mellitus (DM) in obese and non-obese individuals (adjusted for age, sex, smoking status, alcohol drinking status, family history of diabetes, hypertriglyceridemia, high low-density lipoprotein [LDL], low high-density lipoprotein [HDL], hypertension, physical activity, and area). *P* values for difference were derived from multiple logistic regression analysis. Bars represent 95% confidence intervals.

The results of the relationship between the combination of ALT and GGT levels and the prevalence of DM in obese and non-obese individuals are presented in Figure 2. In multivariate analysis, high ALT and/or high GGT levels were significantly associated with the prevalence of DM, even in non-obese groups [non-obese, low ALT, and high GGT levels (OR 1.27; 95% CI, 1.11–1.45); non-obese, high ALT, and low GGT levels (OR 2.06; 95% CI, 1.80–2.36); and non-obese, high ALT and GGT levels (OR 2.19; 95% CI, 1.89–2.52)]. The obese group with both high ALT and GGT levels had the highest OR for the prevalence of DM (OR 4.06; 95% CI, 3.61–4.56). In addition, the obese group with both low ALT and GGT levels was significantly associated with the prevalence of DM, compared to the non-obese group with both low ALT and GGT levels. (OR 1.99; 95% CI, 1.85–2.14).

In addition, when we stratified our analysis by sex (male and female), drinking status (never drinker, ex-drinker, and current drinker), and residential area (Miyagi Prefecture and Iwate Prefecture), the results did not change essentially for all stratified analyses. Excluding participants with a history of hepatitis C or hepatitis B did not alter the results (data not shown).

## DISCUSSION

We showed that the combination of ALT and GGT levels was significantly associated with the prevalence of DM, regardless of obesity. In particular, we showed that the combined ALT and GGT levels were significantly associated with the prevalence of DM, even in the non-obese groups. We also showed that the obese group with both high ALT and GGT levels had the highest OR for the prevalence of DM, compared to the non-obese group with both low ALT and GGT levels. The obese group with both low ALT and GGT levels was significantly associated with the prevalence of DM, compared to the non-obese group with both low ALT and GGT levels.

Previous studies have reported that ALT and GGT levels are associated with the development of DM, regardless of BMI.^11^^–^^19^ Our findings are consistent with these studies. Fatty liver may explain the reason behind the relationship between the combined ALT and GGT levels and the prevalence of DM. The ALT enzyme is present mainly in the cytosol of the liver.^9^ The GGT enzyme is present mainly in the epithelial cells and bile canaliculi.^10^ The ALT and GGT levels are elevated in individuals with fatty liver.^9^^,^^10^ Fatty liver is strongly associated with insulin resistance.^25^^,^^26^ Initial insulin resistance and fatty acid released from the adipocytes promote hepatic steatosis.^25^ Increased fatty acid flux through the liver promotes hepatic gluconeogenesis, worsening hepatic insulin resistance, and potentially worsening whole-body insulin resistance with adverse changes in cardiometabolic risk factors.^25^

In this study, high levels of ALT and GGT were associated with DM prevalence, even in non-obese individuals. Non-obese individuals with elevated GGT levels have been reported to have a higher risk for DM than non-obese individuals with low levels of GGT.^15^ Moreover, another study reported that non-obese participants with elevated ALT and GGT levels had a higher risk for DM than non-obese participants with low ALT and GGT levels.^16^ The findings of our study are consistent with the results reported previously. However, the previous research included only male participants. Thus, as our study used a cross-sectional study design, we included both male and female participants, and showed that the combination of ALT and GGT levels was significantly associated with the prevalence of DM in non-obese individuals. Furthermore, we showed that the obese group had a significantly higher OR for the prevalence of DM, even in the subgroups with low ALT and GGT levels, than in the non-obese group of participants with low levels of both ALT and GGT. Therefore, we confirmed the impact of obesity on the prevalence of DM.

In addition, our results showed that liver function did not change when stratified analysis was performed for alcohol drinking status. Therefore, our findings were applicable to non-drinking subjects; that is, our results cannot be explained by drinking habits. Similarly, our results were consistent when we excluded the participants with hepatitis B or hepatitis C. Thus, our results are also applicable to participants without a history of hepatitis.

Our study has some limitations. First, the study was cross-sectional, so inferences regarding the direction of relationships and/or causality were not possible. Previous studies have reported the direction of relationships and/or causality between ALT and GGT levels and DM.^11^^–^^19^ However, no studies have compared the combination of ALT and GGT levels in obese and non-obese individuals. In the future, prospective cohort studies are required to examine the combined associations of ALT and GGT levels with the development of DM in obese and non-obese individuals. Second, although we adjusted for a substantial number of potential confounders to obtain our results, we may not have included all the relevant confounders. We did not adjust for dietary patterns, so the impact of dietary pattern on the relationship could not be evaluated. However, we believe these points should not be critical.

In conclusion, high ALT and GGT levels are associated with the prevalence of DM, even in non-obese and obese individuals. Obesity is significantly associated with the prevalence of DM, despite low ALT and GGT levels, compared to the non-obese group with both low ALT and GGT levels. This study suggests that correcting ALT and GGT levels and controlling obesity are important for the prevention of DM.
